# Supplementary material for: Comparative anatomy of the middle ear in some lizard species with comments on the evolutionary changes within Squamata
Source: PeerJ. 2021 Jul 22;9:e11722. doi: 10.7717/peerj.11722 (PMC8310623; doi:10.7717/peerj.11722)
Supplement: Supplemental Information 1 [file peerj-09-11722-s001.docx]

**Voucher specimen information**

**GEKKOTA**.

**Gekkonidae**: *Hemidactylus brasilianus* MZUSP 75352; *Phelsuma* *madagascariensis* MZUSP 36938.

**Phyllodactylidae**: *Tarentola mauritanica* MZUSP 44641; *Thecadactylus rapicauda* MZUSP 97833.

**Pygopodidae**: *Lialis jicari* MZUSP 67148. **Sphaerodactylidae**: *Gonatodes albogularis* MUJ 665; *G. concinnatus* MUJ 733.

**IGUANIA. Agamidae**: *Acanthocercus atricollis* MZUSP 95703; *Leiolepis belliana* MZUSP 95700; *Stellagama stellio* MZUSP 95176. **Dactyloidae:** *Anolis antonii* MUJ 376, 384. *A. auratus* MUJ 503, 590. *A. chrysolepis* MUJ 242-243. *A. fuscoauratus* MUJ 746. *A. maculiventris* ICN 4388; MHUA 10466, 10468; MUJ 393. *A. mariarum* ICN 5808; MHUA 10013-10014. *A. tolimensis* MUJ 159, 1234. *A. trachyderma* ICN 8613, 8633. *A. ventrimaculatus* MHUA 10671-10672; MUJ 338. **Hoplocercidae:** *Hoplocercus* *spinosus* MZUSP 92161; *Morunasaurus groi* ICN 6270. **Tropiduridae:** *Stenocercus* *erythrogaster* ICN 9096; *S. trachycephalus* ICN 6261, MUJ 635; *Tropidurus* *pinima* MZUSP 92140.

**SCINCOMORPHA**. **Scincidae:** *Mabuya nigropunctata* ICN 8057, 11249; *M. falconensis* ICN 11312; *Mabuya* sp. 1 ICN 2324, 11242; *Mabuya* sp. 2 ICN 4311, 4332, 7220.

**LACERTOIDEA**. **Gymnophthalmidae:** *Anadia bogotensis* ICN 2178, 2987, 4516, 9868; *Gelanesaurus cochranae* ICN 9453; *Loxopholis rugiceps* ICN 637; *Neusticurus medemi* ICN 8071; *Pholidobolus* *montium* ICN 5603, 5609. *P. vertebralis* ICN 5719; *Riama striata* ICN 9761, ICN 2373, MUJ 653; *Tretioscincus bifasciatus* ICN 5588. **Teiidae:** *Cnemidophorus lemniscatus* ICN 5536. **Lacertidae:** *Acanthodactylus schmidti* MZUSP 95156.
