# Supplementary material for: Comparative anatomy of the middle ear in some lizard species with comments on the evolutionary changes within Squamata
Source: PeerJ. 2021 Jul 22;9:e11722. doi: 10.7717/peerj.11722 (PMC8310623; doi:10.7717/peerj.11722)
Supplement: Supplemental Information 2 — To simplify the tree and to include the species studied, the species from the same genus with the same character state, these were collapsed in a single terminal with the genus name. (*) Species examined in this study. [file peerj-09-11722-s002.docx]

To simplify the tree and to include the species studied, the species from the same genus with the same character state, these were collapsed in a single terminal with the genus name. (*****) Species examined in this study.

| **Terminal taxon** | **Taxa included**  **in the terminal taxon** | **Family** |
| --- | --- | --- |
| *Sphenodon punctatus* | *Sphenodon punctatus* | Sphenodontidae |
| Dibamidae |  | Dibamidae |
| Carphodactylidae |  | Carphodactylidae |
| *Lialis* | *Lialis jicari** | Pygopodidae |
|  | *Lialis burtonis* |  |
| *Aprasia* | *Aprasia* |  |
| Diplodactylidae |  | Diplodactylidae |
| *Coleonyx variegatus* | *Coleonyx variegatus* | Eublepharidae |
| *Eublepharis macularius* | *Eublepharis macularius* |  |
| *Teratoscincus scincus* | *Teratoscincus scincus* | Sphaerodactylidae |
| *Gonatodes* | *Gonatodes albogularis** |  |
|  | *Gonatodes concinnatus** |  |
| *Thecadactylus rapicauda* | *Thecadactylus rapicauda** | Phyllodactylidae |
| *Tarentola mauritanica* | *Tarentola mauritanica** |  |
| *Gekko gecko* | *Gekko gecko* | Gekkonidae |
| *Hemidactylus brasilianus* | *Hemidactylus brasilianus** |  |
| *Phelsuma madagascariensis* | *Phelsuma madagascariensis** |  |
| *Chondrodactylus bibronii* | *Chondrodactylus bibronii* |  |
| *Xantusia henshawi* | *Xantusia henshawi* | Xantusiidae |
| *Lepidophyma* | *Lepidophyma flavimaculatum* |  |
|  | *Lepidophyma smithi* |  |
| Gerrhosauridae |  | Gerrhosauridae |
| Cordylidae |  | Cordylidae |
| *Eutropis multifasciata* | *Eutropis multifasciata* | Scincidae |
| *Mabuya nigropunctatum* | *Mabuya nigropunctatum** |  |
| *Mabuya spp.* | *sp. 1 y sp. 2** |  |
| *Mabuya falconensis* | *Mabuya falconensis** |  |
| *Trachylepis brevicollis* | *Trachylepis brevicollis* |  |
| *Tupinambis teguixin* | *Tupinambis teguixin* | Teiidae |
| *Cnemidophorus lemniscatus* | *Cnemidophorus lemniscatus** |  |
| *Pholidoscelis lineolatus* | *Pholidoscelis lineolatus* |  |
| *Alopoglossidae* |  | Alopoglossidae |
| *Anadia bogotensis* | *Anadia bogotensis** | Gymnophthalmidae |
| *Gelanesaurus cochranae* | *Gelanesaurus cochranae** |  |
| *Loxopholis rugiceps* | *Loxopholis rugiceps** |  |
| *Riama striata* | *Riama striata** |  |
| *Neusticurus medemi* | *Neusticurus medemi** |  |
| *Pholidobolus* | *Pholidobolus montium** |  |
|  | *Pholidobolus vertebralis** |  |
| *Tretioscincus bifasciatus* | *Tretioscincus bifasciatus** |  |
| *Rhineura floridana* | *Rhineura floridana* | Rhineuridae |
| *Bipes biporus* | *Bipes biporus* | Bipedidae |
| *Blanus* | *Blanus* | Blanidae |
| Cadeidae |  | Cadeidae |
| *Trogonophidae* | *Diplometopon zarudnyi* | Trogonophidae |
|  | *Trogonophis wiegmanni* |  |
| Amphisbaenidae | *Amphisbaena alba* | Amphisbaenidae |
|  | *Amphisbaena darwini trachura* |  |
|  | *Amphisbaena fuliginosa* |  |
|  | *Amphisbaena manni* |  |
|  | *Chirindia langi* |  |
|  | *Cynisca leucura* |  |
|  | *Monopeltis c. capensis* |  |
|  | *Zygaspis violacea* |  |
| *Acanthodactylus cf. schmidti* | *Acanthodactylus cf. schmidti** | Lacertidae |
| *Podarcis muralis* | *Podarcis muralis* |  |
| *Timon lepidus* | *Timon lepidus* |  |
| *Heloderma suspectum* | *Heloderma suspectum* | Helodermatidae |
| *Xenosaurus grandis* | *Xenosaurus grandis* | Xenosauridae |
| Diploglossidae |  | Diploglossidae |
| *Anniella pulchra* | *Anniella pulchra* | Anguidae |
| *Anguis fragilis* | *Anguis fragilis* |  |
| Shinisauridae |  | Shinisauridae |
| *Lanthanotus borneensis* | *Lanthanotus borneensis* | Lanthanotidae |
| *Varanus* | *Varanus bengalensis* | Varanidae |
|  | *Varanus niloticus* |  |
|  | *Varanus salvator* |  |
| Chamaeleo | Chamaeleo | Chamaeleonidae |
| *Uromastyx aegyptia* | *Uromastyx aegyptia* | Agamidae |
| *Broncochela jubata* | *Broncochela jubata* |  |
| *Leiolepis belliana* | *Leiolepis belliana* |  |
| *Stellagama stellio* | *Stellagama stellio** |  |
| *Phrynocephalus maculatus* | *Phrynocephalus maculatus* |  |
| *Phrynocephalus sp.* | *Phrynocephalus sp.* |  |
| *Acanthocercus atricollis* | *Acanthocercus atricollis** |  |
| *Draco volans* | *Draco volans* |  |
| *Ceratophora* | *Ceratophora stoddartii* |  |
|  | *Ceratophora tennenti* |  |
| Leiocephalidae |  | Leiocephalidae |
| *Iguana iguana* |  | Iguanidae |
| *Hoplocercus sp.* | *Hoplocercus* sp.* | Hoplocercidae |
| *Morunasaurus groi* | *Morunasaurus groi ** |  |
| *Corytophanidae* |  | Corytophanidae |
| *Crotaphytus collaris* |  | Crotaphytidae |
| *Stenocercus* | *Stenocercus erythrogaster** | Tropiduridae |
|  | *Stenocercus trachycephalus** |  |
| *Tropidurus pinima* | *Tropidurus pinima** |  |
| Opluridae |  | Opluridae |
| Leiosauridae |  | Leiosauridae |
| Liolaemidae |  | Liolaemidae |
| *Cophosaurus texanus* | *Cophosaurus texanus* | Phrynosomatidae |
| *Callisaurus* | *Callisaurus draconoides* |  |
| *Holbrookia maculata* | *Holbrookia maculata* |  |
| *Phrynosoma* | *Phrynosoma coronatum* |  |
|  | *Phrynosoma platyrhinos* |  |
| *Sceloporus magister* | *Sceloporus magister* |  |
| Polychotidae |  | Polychotidae |
| *Anolis* | *Anolis antonii** | Dactyloidae |
|  | *Anolis auratus** |  |
|  | *Anolis chrysolepis** |  |
|  | *Anolis fuscoauratus** |  |
|  | *Anolis maculiventris** |  |
|  | *Anolis mariarum** |  |
|  | *Anolis tolimensis** |  |
|  | *Anolis trachyderma** |  |
|  | *Anolis ventrimaculatus** |  |
| Serpentes | Clade Snakes |  |
