# Supplementary material for: Comparative anatomy of the middle ear in some lizard species with comments on the evolutionary changes within Squamata
Source: PeerJ. 2021 Jul 22;9:e11722. doi: 10.7717/peerj.11722 (PMC8310623; doi:10.7717/peerj.11722)
Supplement: Supplemental Information 3 — Posterior probabilities estimated for each node by the Bayesian Ancestral State Reconstructions modelled using the models with all rates different (ARD) and equal rates (ER). The higher values in bold. [file peerj-09-11722-s003.docx]

Posterior probabilities estimated for each node by the Bayesian Ancestral State Reconstructions modelled using the models with all rates different (ARD) and equal rates (ER). The higher values in bold.

Character 1. Length of the columella concerning that of the extracolumella central axis.

States: equal length, longer, and shorter.

| **Character 1 ARD model** | | | | | **Character 1 ER model** | | | |
| --- | --- | --- | --- | --- | --- | --- | --- | --- |
| node | **inapplicable** | **equal** | **longer** | **shorter** | **inapplicable** | **equal** | **longer** | **shorter** |
| 2 | 0.125 | 0.31250007 | **0.33333325** | 0.22916667 | 0.25 | 0.25 | 0.25 | 0.25 |
| 4 | 0.125 | 0.31250007 | **0.33333325** | 0.22916667 | 0.25 | 0.25 | 0.25 | 0.25 |
| 7 | 0.12500008 | 0.31249988 | **0.33333298** | 0.22916706 | **0.25000001** | 0.24999999 | 0.24999999 | **0.25000001** |
| 17 | 0.13653076 | 0.30545291 | **0.32404204** | 0.23397429 | 0.25 | 0.25 | 0.25 | 0.25 |
| 18 | 0.12500076 | 0.31249893 | **0.33333187** | 0.22916844 | 0.25 | 0.25 | 0.25 | 0.25 |
| 19 | 0.125186 | 0.31233201 | **0.33311723** | 0.22936476 | 0.25 | 0.25 | 0.25 | 0.25 |
| 20 | 0.125 | 0.31250007 | **0.33333325** | 0.22916667 | 0.25 | 0.25 | 0.25 | 0.25 |
| 25 | 0.12738603 | 0.31080407 | **0.33113174** | 0.23067817 | 0.25 | 0.25 | 0.25 | 0.25 |
| 26 | 0.12698867 | 0.31106573 | **0.33145486** | 0.23049073 | 0.25 | 0.25 | 0.25 | 0.25 |
| 27 | 0.125 | 0.31250007 | **0.33333325** | 0.22916667 | 0.25 | 0.25 | 0.25 | 0.25 |
| 29 | 0.13653284 | 0.30554922 | **0.32390936** | 0.23400858 | 0.25 | 0.25 | 0.25 | 0.25 |
| 30 | 0.12621074 | 0.31158184 | **0.33214639** | 0.23006103 | 0.25 | 0.25 | 0.25 | 0.25 |
| 33 | 0.12500075 | 0.31249895 | **0.33333188** | 0.22916842 | 0.25 | 0.25 | 0.25 | 0.25 |
| 34 | 0.12897694 | 0.30983815 | **0.32980322** | 0.23138168 | 0.25 | 0.25 | 0.25 | 0.25 |
| 35 | 0.125 | 0.31250007 | **0.33333325** | 0.22916667 | 0.25 | 0.25 | 0.25 | 0.25 |
| 38 | 0.13129247 | 0.30842719 | **0.32799604** | 0.2322843 | 0.25 | 0.25 | 0.25 | 0.25 |
| 39 | 0.125 | 0.31250007 | **0.33333325** | 0.22916667 | 0.25 | 0.25 | 0.25 | 0.25 |
| 42 | 0.1536286 | 0.29633163 | **0.31118633** | 0.23885345 | 0.25 | 0.25 | 0.25 | 0.25 |
| 43 | 0.125 | 0.31250007 | **0.33333325** | 0.22916667 | 0.25 | 0.25 | 0.25 | 0.25 |
| 47 | 0.12907949 | 0.30977633 | **0.32971997** | 0.2314242 | 0.25 | 0.25 | 0.25 | 0.25 |
| 50 | 0.12524732 | 0.31228275 | **0.33305825** | 0.22941168 | 0.25 | 0.25 | 0.25 | 0.25 |
| 51 | 0.125 | 0.31250007 | **0.33333325** | 0.22916667 | 0.25 | 0.25 | 0.25 | 0.25 |
| 55 | 0.12500131 | 0.3124982 | **0.33333096** | 0.22916953 | 0.25 | 0.25 | 0.25 | 0.25 |
| 60 | 0.125 | 0.31250007 | **0.33333325** | 0.22916667 | 0.25 | 0.25 | 0.25 | 0.25 |
| 73 | 0.12500002 | 0.31250003 | **0.33333321** | 0.22916674 | 0.25 | 0.25 | 0.25 | 0.25 |

Character 2. Extracolumella,

States: reduced, expanded, absent, extensive.

|  | **Character 2 ARD model** | | | | **Character 2 ER model** | | | |
| --- | --- | --- | --- | --- | --- | --- | --- | --- |
| node | **absent** | **expanded** | **extensive** | **reduced** | **absent** | **expanded** | **extensive** | **reduced** |
| 2 | 0.00941888 | 0.03648943 | 0.00039059 | **0.9537011** | 0.00246515 | 0.06506425 | 0.0023549 | **0.9301157** |
| 4 | 0.15257646 | **0.82442096** | 0.00636789 | 0.01663468 | 0.00322959 | **0.98529321** | 0.00058526 | 0.01089194 |
| 7 | 0.14413976 | **0.84765478** | 0.00696372 | 0.00124174 | 0.08343365 | **0.91055783** | 0.00233453 | 0.003674 |
| 17 | 0.00446556 | 0.00653912 | 4.7349E-06 | **0.98899059** | 0.00036623 | 0.04194958 | 0.00031268 | **0.9573715** |
| 18 | 0.00638139 | 0.03082852 | 0.00081547 | **0.96197462** | 0.00223274 | 0.05996045 | 0.00219596 | **0.93561085** |
| 19 | 0.02824684 | 0.1207859 | 0.01240961 | **0.83855764** | 0.01072824 | 0.15947383 | 0.01069638 | **0.81910155** |
| 20 | 0.14404787 | **0.8049389** | 0.01589714 | 0.0351161 | 0.00167592 | **0.98004529** | 0.00167533 | 0.01660346 |
| 25 | 0.00014604 | 0.00026864 | 7.5186E-08 | **0.99958524** | 9.172E-05 | 0.00381466 | 1.5214E-05 | **0.99607841** |
| 26 | 2.3585E-05 | 6.8293E-05 | 2.2619E-05 | **0.9998855** | 0.00025523 | 0.00247836 | 0.00012663 | **0.99713979** |
| 27 | 8.9917E-06 | 1.6726E-05 | 3.2974E-08 | **0.99997425** | 1.0904E-05 | 1.5188E-05 | 1.0657E-05 | **0.99996325** |
| 29 | 4.4891E-05 | 5.8764E-05 | 1.1675E-06 | **0.99989518** | 3.8742E-05 | 4.1769E-05 | 3.8567E-05 | **0.99988092** |
| 30 | 9.0925E-06 | 2.1004E-05 | 1.1744E-07 | **0.99996979** | 1.2475E-05 | 1.2825E-05 | 1.2455E-05 | **0.99996225** |
| 33 | 6.4069E-05 | 0.0001528 | 0.00380144 | **0.99598169** | 0.00640005 | 0.00485359 | 0.00373686 | **0.9850095** |
| 34 | 1.6843E-05 | 4.134E-05 | 0.00891021 | **0.9910316** | 0.0155652 | 0.00484359 | 0.00905005 | **0.97054116** |
| 35 | 0.0001023 | 0.00030638 | **0.94305363** | 0.0565377 | **0.58302408** | 0.00444698 | 0.32237955 | 0.09014939 |
| 38 | 2.3929E-07 | 4.1413E-07 | **0.95923663** | 0.04076271 | 0.26235096 | 0.00309711 | **0.7031893** | 0.03136263 |
| 39 | 0.01472645 | 0.06951915 | 0.00229906 | **0.91345534** | 0.00519376 | 0.07391632 | 0.00454882 | **0.9163411** |
| 42 | 0.00020426 | 0.00037516 | 1.0146E-06 | **0.99941956** | 0.00010956 | 0.00342768 | 2.0399E-05 | **0.99644236** |
| 43 | 2.0019E-05 | 5.2386E-05 | 1.9831E-07 | **0.9999274** | 2.9607E-05 | 5.7883E-05 | 2.8847E-05 | **0.99988366** |
| 47 | 3.2805E-05 | 9.7245E-05 | 4.9062E-07 | **0.99986946** | 6.311E-05 | 6.3144E-05 | 6.3109E-05 | **0.99981064** |
| 50 | 0.00148325 | 0.00493572 | 8.2551E-05 | **0.99349848** | 0.00043424 | 0.00907955 | 0.00037518 | **0.99011104** |
| 51 | 6.2213E-05 | 0.00018636 | 4.8397E-07 | **0.99975095** | 3.7202E-05 | 0.00066364 | 3.6056E-05 | **0.9992631** |
| 55 | 0.00103279 | 0.00273171 | 2.3084E-05 | **0.99621241** | 0.00022176 | 0.00383105 | 0.00022176 | **0.99572543** |
| 60 | 0.14711138 | **0.79982811** | 0.00464735 | 0.04841316 | 0.00300624 | **0.94598549** | 0.00300355 | 0.04800472 |
| 73 | 0.18275532 | **0.60568949** | 0.00071967 | 0.21083552 | 0.00092302 | **0.91419553** | 0.00092302 | 0.08395842 |

Character 3. Internal Process,

States: absent, present.

|  | **Character 3 ARD model** | | | **Character 3 ED model** | | |
| --- | --- | --- | --- | --- | --- | --- |
| node | **inapplicable** | **absent** | **present** | **inapplicable** | **absent** | **present** |
| 2 | 0 | 0 | **1** | 0.00560688 | 0.35811667 | **0.63627646** |
| 4 | 0 | **0.9854981** | 0.0145019 | 0.00280219 | **0.98870369** | 0.00849412 |
| 7 | 0 | **0.98589652** | 0.01410348 | 0.08946998 | **0.90665117** | 0.00387884 |
| 17 | 0 | 0 | **1** | 0.00065542 | 0.20546221 | **0.79388237** |
| 18 | 0 | 0 | **1** | 0.00093644 | 0.21261191 | **0.78645165** |
| 19 | 0 | 0 | **1** | 0.00111202 | 0.18135747 | **0.81753051** |
| 20 | 0 | 0 | **1** | 0.00428048 | 0.23578752 | **0.75993199** |
| 25 | 0 | 0 | **1** | 0.00107607 | 0.02129052 | **0.97763341** |
| 26 | 0 | 0 | **1** | 0.00272188 | 0.02078862 | **0.9764895** |
| 27 | 0 | 0 | **1** | 0.00120783 | 0.08835176 | **0.91044042** |
| 29 | 0 | 0.26917173 | **0.73082827** | 0.00347792 | 0.34756981 | **0.64895227** |
| 30 | 0 | **0.80846955** | 0.19153045 | 0.00117945 | **0.91574372** | 0.08307684 |
| 33 | 0 | 0 | **1** | 0.03488042 | 0.0144239 | **0.95069568** |
| 34 | 0.10509206 | 0.01670499 | **0.87820295** | 0.1300311 | 0.01824747 | **0.85172143** |
| 35 | **0.92984702** | 0.01362405 | 0.05652893 | **0.89092171** | 0.00726564 | 0.10181265 |
| 38 | **0.92865237** | 0.01465475 | 0.05669288 | **0.88559842** | 0.01301169 | 0.10138989 |
| 39 | 0 | 0 | **1** | 0.00055168 | 0.00030564 | **0.99914267** |
| 42 | 0 | 0 | **1** | 0.00034968 | 0.0030405 | **0.99660983** |
| 43 | 0 | 0 | **1** | 4.6786E-05 | 0.00010975 | **0.99984347** |
| 47 | 0 | 0 | **1** | 0.00158931 | 0.02913395 | **0.96927674** |
| 50 | 0 | 0 | **1** | 0.00010575 | 0.00060191 | **0.99929235** |
| 51 | 0 | 0 | **1** | 2.8163E-05 | 3.6819E-05 | **0.99993502** |
| 55 | 0 | 0 | **1** | 1.9847E-05 | 1.9848E-05 | **0.99996031** |
| 60 | 0 | 0 | **1** | 0.00025916 | 0.00028217 | **0.99945867** |
| 73 | 0 | 0 | **1** | 1.6068E-05 | 1.6068E-05 | **0.99996786** |
