# Supplementary figures and images for: Comparative anatomy of the middle ear in some lizard species with comments on the evolutionary changes within Squamata
Source: PeerJ. 2021 Jul 22;9:e11722. doi: 10.7717/peerj.11722 (PMC8310623; doi:10.7717/peerj.11722)

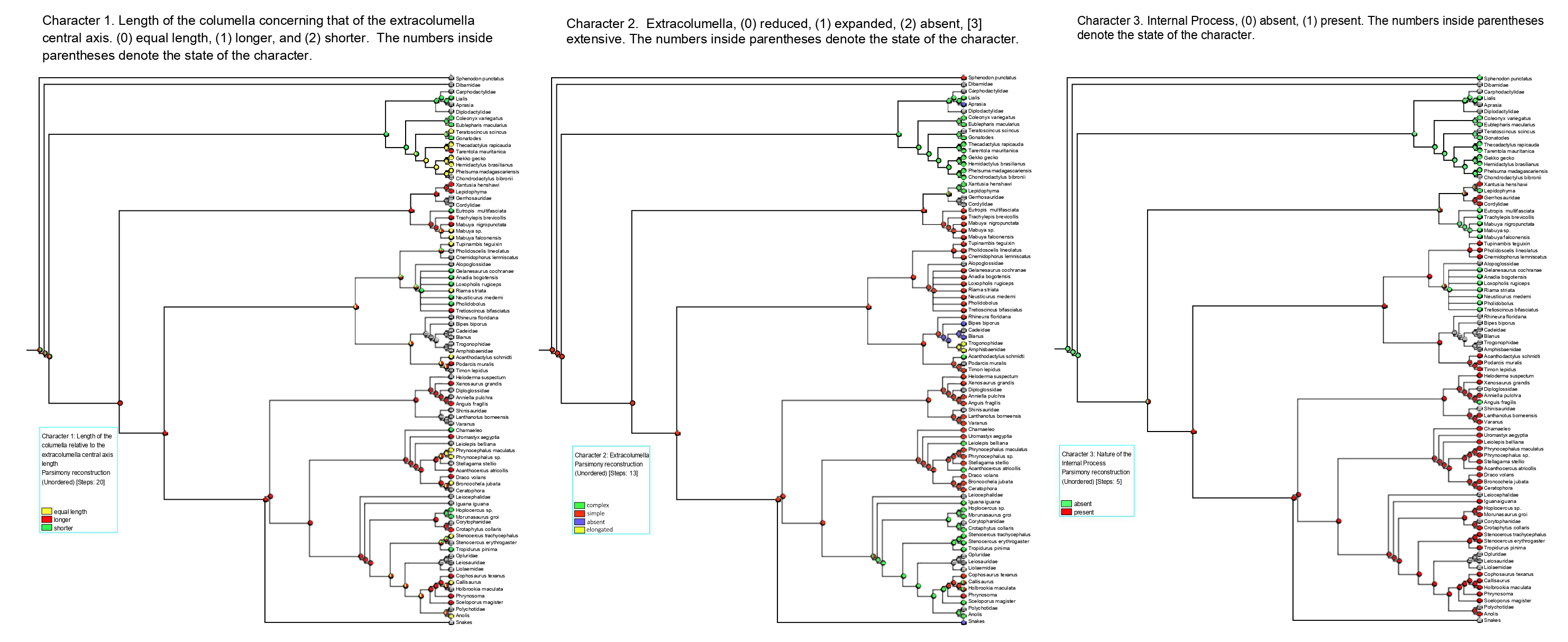

Supplement: Supplemental Information 4 [file peerj-09-11722-s004.png]

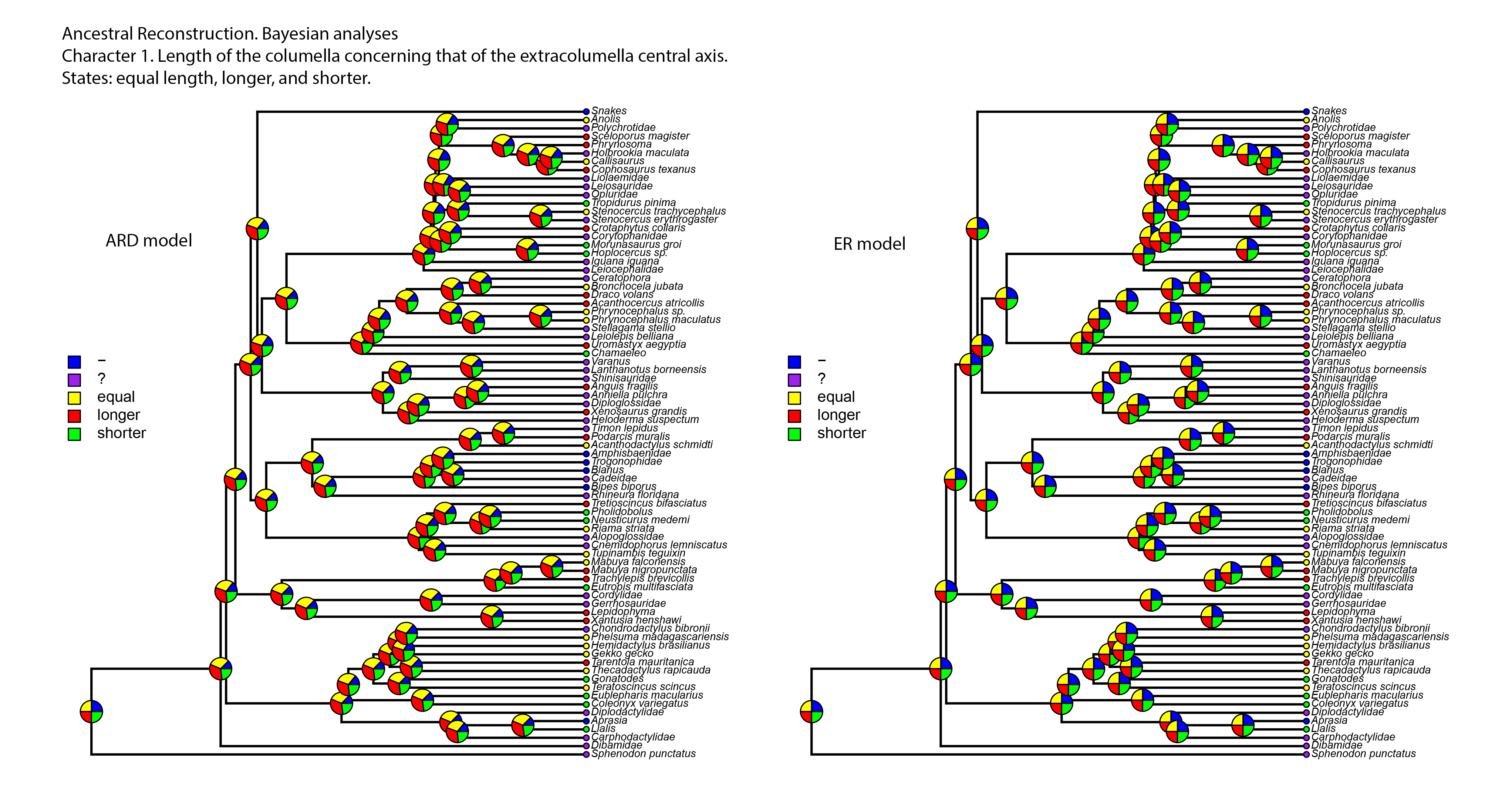

Supplement: Supplemental Information 5 [file peerj-09-11722-s005.png]

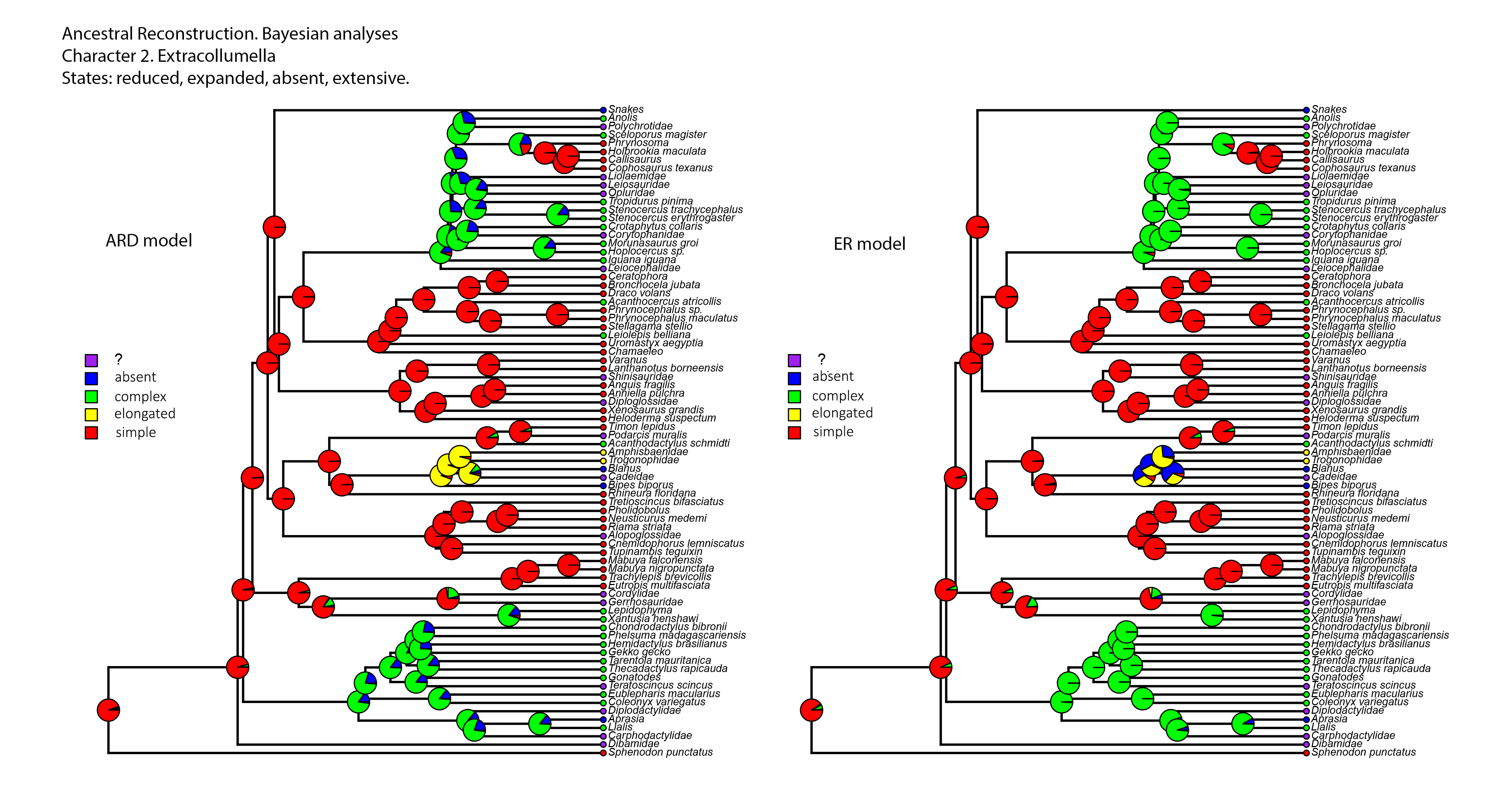

Supplement: Supplemental Information 6 [file peerj-09-11722-s006.png]

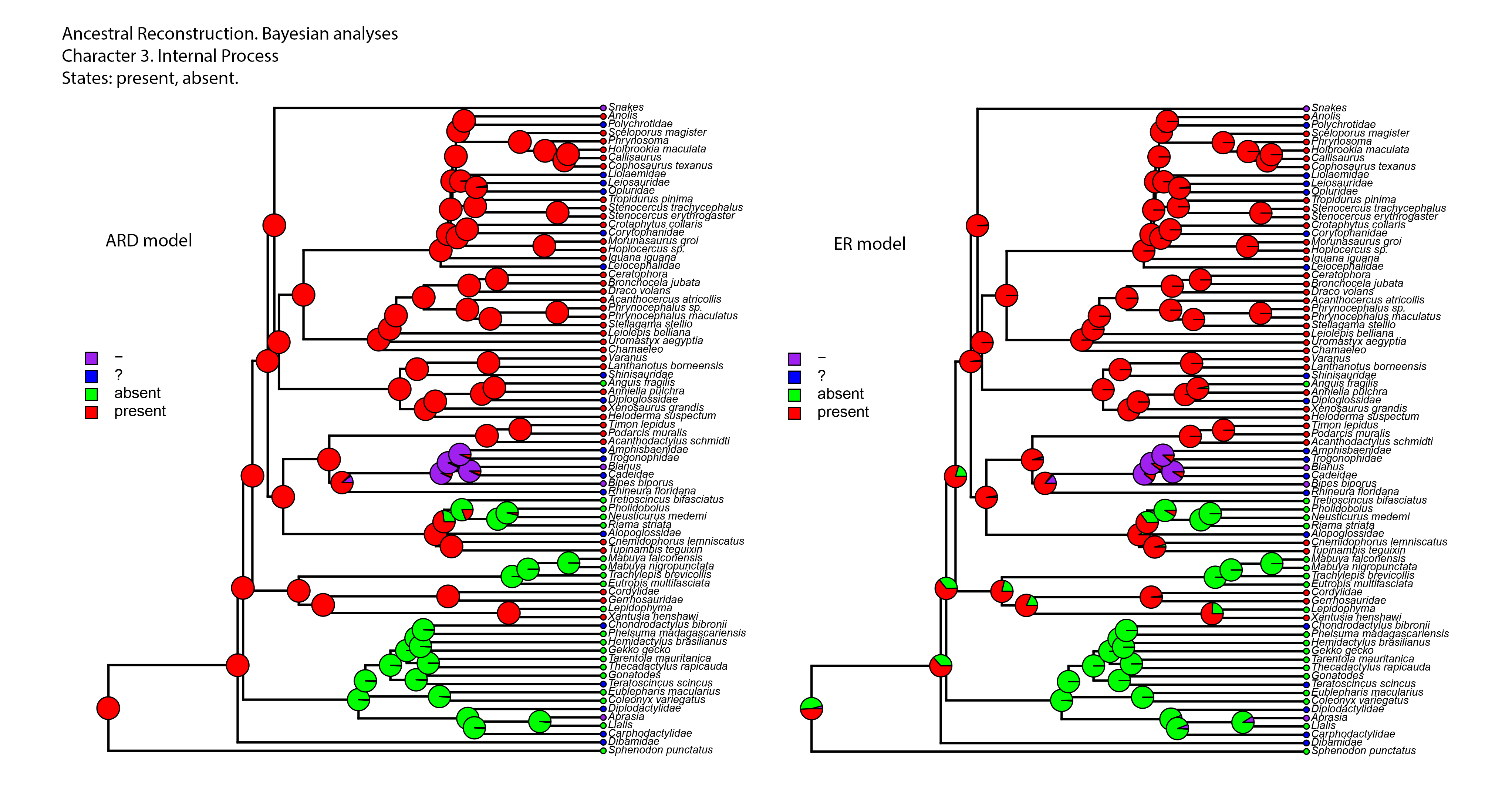

Supplement: Supplemental Information 7 [file peerj-09-11722-s007.png]
